# A Novel Chordoma Xenograft Allows *In Vivo* Drug Testing and Reveals the Importance of NF-κB Signaling in Chordoma Biology

**DOI:** 10.1371/journal.pone.0079950

**Published:** 2013-11-06

**Authors:** Matteo M. Trucco, Ola Awad, Breelyn A. Wilky, Seth D. Goldstein, Ruili Huang, Robert L. Walker, Preeti Shah, Varalakshmi Katuri, Naheed Gul, Yuelin J. Zhu, Edward F. McCarthy, Ido Paz-Priel, Paul S. Meltzer, Christopher P. Austin, Menghang Xia, David M. Loeb

**Affiliations:** 1 Sidney Kimmel Comprehensive Cancer Center, Johns Hopkins University, Baltimore, Maryland, United States of America; 2 National Center for Advancing Translational Sciences, NIH, Rockville, Maryland, United States of America; 3 Genetics Branch, Center for Cancer Research, National Cancer Institute, NIH, Bethesda, Maryland, United States of America; 4 Department of Pathology, Johns Hopkins University, Baltimore, Maryland, United States of America; Philipps University, Germany

## Abstract

Chordoma is a rare primary bone malignancy that arises in the skull base, spine and sacrum and originates from remnants of the notochord. These tumors are typically resistant to conventional chemotherapy, and to date there are no FDA-approved agents to treat chordoma. The lack of *in vivo* models of chordoma has impeded the development of new therapies for this tumor. Primary tumor from a sacral chordoma was xenografted into NOD/SCID/IL-2R γ-null mice. The xenograft is serially transplantable and was characterized by both gene expression analysis and whole genome SNP genotyping. The NIH Chemical Genomics Center performed high-throughput screening of 2,816 compounds using two established chordoma cell lines, U-CH1 and U-CH2B. The screen yielded several compounds that showed activity and two, sunitinib and bortezomib, were tested in the xenograft. Both agents slowed the growth of the xenograft tumor. Sensitivity to an inhibitor of IκB, as well as inhibition of an NF-κB gene expression signature demonstrated the importance of NF-κB signaling for chordoma growth. This serially transplantable chordoma xenograft is thus a practical model to study chordomas and perform *in vivo* preclinical drug testing.

## Introduction

Chordoma is a rare primary bone malignancy, accounting for 1-4% of all bone tumors, which is believed to arise from remnants of the notochord. Chordomas are typically found in the clivus, sacrum or spine, with a near equal distribution among the three locations. There are three histologic subtypes of chordoma: classical, chondroid, and dedifferentiated, though there is significant overlap between these three types and a single chordoma can exhibit regions of different histology [1]. Though often low-grade, these tumors tend to be locally invasive with a high rate of recurrence. This behavior, especially in areas where complete resection with negative margins can be difficult, if not impossible, frequently results in a prolonged and difficult clinical course with poor outcomes [2]. 

Contemporary management of chordoma primarily consists of surgical resection and radiation therapy. However, because these tumors arise along the axial skeleton, complete surgical resection and delivery of definitive radiation is often not feasible. Conventional chemotherapeutic agents are not effective [3]. To date, the most effective agent against chordoma is imatinib, which in a phase II study demonstrated a clinical benefit rate of 64%, though median progression free survival was only 9 months [4]. There are still no FDA-approved agents for the treatment of chordoma.

There is limited understanding of the important signaling pathways in chordoma. Some hints have been gleaned from evaluation of pathologic specimens. For example, analysis of 42 chordoma specimens using immunohistochemistry, fluorescence *in situ* hybridization, and a phospho-kinase antibody array demonstrated that epidermal growth factor receptor and AKT are frequently activated in this tumor [5]. A similar approach also implicated the platelet-derived growth factor receptor and the mTOR pathway [6]. Brachyury, a transcription factor expressed in the developing notochord that is used as a diagnostic marker of chordoma [7], also plays a critical role in chordoma biology. Duplication of this locus is associated with familial chordoma, and this locus is amplified in 5% of sporadic tumors [8,9], and knock-down of brachyury expression in the JHC7 cell line inhibits proliferation [10]. A recent study using integrated functional genomics identified several target genes of brachyury [11], suggesting that the mechanism of chordoma formation involves multiple signaling pathways.

Limited *in vitro* models and a lack of practical *in vivo* models has hindered the translation of these observations into novel therapies for chordoma patients. In this study, we describe the establishment of a dedifferentiated chordoma xenograft, its use for *in vivo* validation of activity of agents identified by a high throughput *in vitro* screen, and the importance of NF-κB signaling in chordoma biology.

## Materials and Methods

### Establishment of xenograft

All procedures and experiments involving mice were performed according to protocols approved by Johns Hopkins Animal Care and Use Committee (Protocol MO08M301). All surgical procedures were performed using a mixture of xylazine and ketamine, and post-operative pain was managed with carprofen. The use of patient material was approved by the Institutional Review Board of the Johns Hopkins Hospital, and written informed consent was obtained according to institutional standard procedures. A freshly obtained tumor sample was transferred from the operating room to the laboratory, and a single cell suspension was created by mechanical mincing with scalpels and further dissociated with 0.25% trypsin (Wheaton Sciences, USA). The single cell suspension was injected into the parasacral region of NOD/SCID/IL-2Rγ-null (NOG/SCID) mice. The first tumors grew after 3 months. When tumors reached 2cm in diameter, mice were sacrificed and the tumors were harvested, cut into 4 mm fragments, and viably frozen in RPMI 1640 with 10% DMSO (Sigma-Aldrich, USA). For implantation, viably frozen tumor was thawed to 37 °C. Tumor sections were washed three times in RPMI (GIBCO, USA) to remove DMSO. Fragments were then placed in BD Matrigel Matrix (BD Bioscience, USA) and kept on ice until implanted subcutaneously in the flanks of NOG/SCID mice. 

### Histopathological and Immunohistochemical Analyses

Formalin-fixed, paraffin-embedded tissue was used for all histopathological and immunohistochemical analyses. Sections were cut to 5 mm, deparaffinized, and stained with haematoxylin and eosin (Sigma-Aldrich) or processed for immunohistochemistry with antibodies specific for human Brachyury (dilution 1:50, Santa Cruz Biotechnology #SC-374321, A-4; dilution 1:50, Santa Cruz Biotechnology #SC-17745, C-19) or for the p65 subunit of NF-κB (dilution 1:1000, Santa Cruz Biotechnology #SC-109). Samples were deparaffinized in xylene and rehydrated in graded alcohol and rinsed in PBS. Antigens were retrieved by boiling samples for 30 minutes in citrate buffer, pH 6 (Invitrogen). Nonspecific binding sites were blocked using 1 ml PBS containing 5% goat serum and 1% bovine serum albumin (BSA). The sections were incubated overnight at 4 °C in a humidor with the respective monoclonal primary antibody, diluted with 1% goat serum, 0.2% BSA and 0.3% Triton X-100 in PBS (pH 7.4), followed by washing with PBS. For Brachyury, detection of antibody binding was achieved using a fluorescent-conjugated secondary antibody (Molecular Probes, Invitrogen #F2761) with nuclear counterstaining with DAPI (Sigma-Aldrich). For NF-κB, sections were incubated with peroxidase-conjugated secondary antibody (Jackson Immunoresearch Laboratories, USA #323-005-021) of appropriate specificity, 3,39-diaminobenzidine (DAB, Pierce) was used as substrate for peroxidase and counterstaining was performed with modified Harris hematoxylin solution (Sigma-Aldrich). Sections were dehydrated by passage through graded alcohol concentrations and finally xylene. Cover slips were mounted using DPX (Sigma-Aldrich). Completed immunostaining was visualized using a Nikon E600 microscope and photographed with a Nikon DXM1200F digital camera using ACT-1 software.

### Polymerase Chain Reaction (PCR)

RNA was extracted from tissue using RNeasy Mini Kit according to manufacturer’s instructions (QIAGEN Inc, Valencia, CA) and reverse transcribed (Iscript Reverse Transcriptase Bio-Rad, Hercules, CA). For the PCR reaction we used Platinum Blue PCR SuperMix (Invitogen, Grand Island, NY), 1 µl cDNA and 31 cycles of 94 °C for 15 seconds, 60 °C for 30 seconds, 72 °C for 30 seconds. The PCR product was analyzed by 1% agarose gel electrophoresis and visualized with ethidium bromide. The Brachyury primers were, forward: GCC AGA CTG GAG AGT TGA GG; reverse, CAG GTG GTC CAC TCG GTA CT (7). For quantitative PCR, 1 µl cDNA was mixed with 12.5 µl SYBER Green SuperMix and appropriate primers. Quantitative PCR was performed using a standard two-step amplification/melt protocol. Primers for all other RT-PCR experiments were obtained from SABiosciences (Frederick, MD; BCL2- catalog # PPH00079B; BCLx- catalog # PPH00078B; AKT- catalog # PPH00088B; IL6- catalog # PPH00560C; IL8- catalog # PPH00568A; Vimentin-catalog # PPH00417F).

### Whole genome SNP genotyping

Xenograft genomic DNA (750 ng) was processed and hybridized to a HumanCNV370-Duo v1.0 BeadChip according to the manufacturer’s instructions. The chip was scanned on a BeadArray Reader, data extracted using BeadStudio software (Illumina, San Diego, CA), and exported to Nexus Copy Number for graphical display (BioDiscovery, Hawthorne, CA)

### Gene expression profiling

Xenograft total RNA (200 ng) was reverse transcribed, followed by second strand synthesis and *in vitro* transcription incorporating biotinylated dUTP using Illumina TotalPrep RNA Amplification Kit (Ambion Inc, Austin, TX) according to the manufacturer’s instructions. Biotinylated cRNA was hybridized to a HumanRef-8 v2 Expression BeadChip, scanned on a BeadArray Reader, and data extracted using BeadStudio software (Illumina, San Diego, CA). Gene expression data were compared to previously generated expression data from 3 classical chordomas using LIMMA (www.bioconductor.org). Microarray data is available through the Gene Expression Omnibus database under accession numbers GSE50135, GSE50136, and GSE50137.

### High-Throughput Drug Screen

 Chordoma cell lines U-CH1 and U-CH2B were used in the screen and a counter screen was performed on the cell line CCL4, a line previously thought to be composed of chordoma cells, but demonstrated to not express brachyury. A total of 2,816 drugs from the NCGC Pharmaceutical Collection (NPC) [12] were screened (US FDA 1,456, UK/EU/Canada/Japan 634, Investigational 726). Cells were plated and exposed to multiple drug concentrations ranging from 0.5 nM to 46 µM for 48 hours. CellTiter-Glo assay was used to measure cell viability after incubation with drug. Drugs were considered “positive” if they were toxic to U-CH1 and/or U-CH2B cells but not to CCL4. Details of this screen have been recently published [13].

### In vivo drug testing

For drug testing, cohorts of 4 NOG/SCID mice were used. Under sterile conditions, mice were anesthetized with a Ketamine (90 mg/ml) and Xylazine (10 mg/ml) preparation and received 6-10 µl/g via intra-peritoneal injection. An approximately 5 mm incision was made over the right parasacral region and a subcutaneous pocket was created by blunt dissection. A 4x4 mm tumor fragment was placed in the subcutaneous pocket and the incision was closed with a surgical clip. After 10-14 days, surgical clips were removed and treatment was started. Control mice were treated with 100 µl intraperitoneal injections of DMSO, Polyethylene Glycol-200 and PBS (ratio 1:2:2). Sunitinib was administered at a dose of 40 mg/kg/day in DMSO by gavage five days a week. Bortezomib was administered at a dose of 0.7 mg/kg/day in DMSO/PEG/PBS vehicle via intraperitoneal injection on days 0, 4, 11 and then weekly. IMD-0354 was administered at a dose of 5 mg/kg in DMSO/PEG/PBS vehicle 5 days a week via intraperitoneal injection. Mice were monitored daily for tumor growth, and volume was measured every 2-3 days once the tumor became palpable. Mice were sacrificed when tumor diameter reached 2 cm.

### Statistical analysis

 Statistical analysis was performed using Prism v5.0 software (GraphPad Software, Inc., La Jolla, CA). Growth curves were analyzed for significance using a 2-way ANOVA, and gene expression data were analyzed using an unpaired, two-tailed Student t test. In both cases, a p value < 0.05 was considered significant.

## Results

### Establishment of a primary dedifferentiated chordoma xenograft

**Figure 1:**
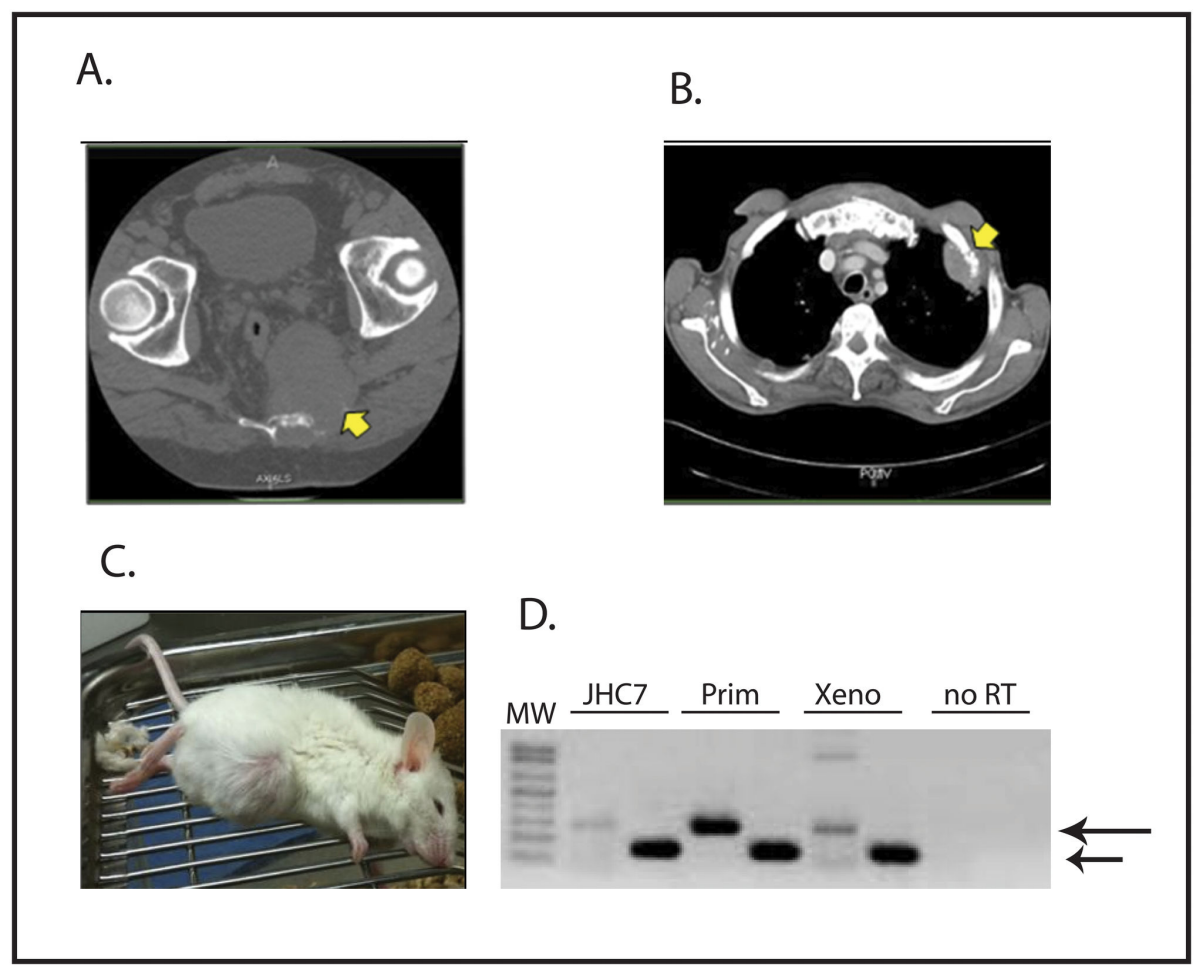
A) CT scan showing patient’s primary sacral chordoma. Tumor is indicated by the arrow. B) CT scan showing the patient’s metastatic relapse to the chest wall (indicated by the arrow). C) Photograph of a mouse bearing the xenograft established from a sample of the patient’s primary tumor. D) RT-PCR showing brachyury expression in xenograft. GAPDH is used as a loading control and control for RNA integrity. RNA from the JHC7 cell line (a kind gift from Dr. A Quinones-Hinojosa, Johns Hopkins University [10]) and a sample from an unrelated patient’s chordoma (Prim) were used as positive controls for brachyury expression, and a sample prepared without reverse transcriptase (no RT) served as a negative control. The upper arrow indicates the brachyury amplicon, and the lower arrow indicates the GAPDH control. The identity of the brachyury band was confirmed by direct sequencing of the PCR product.

A 50 year old man presented to The Johns Hopkins Hospital with sacral pain in 2007 and was diagnosed with sacral chordoma (Figure 1A). Tumor was resected in November 2007 and on pathological evaluation was consistent with classical chordoma. The patient had a metastatic relapse in April 2009 (Figure 1B). Biopsy of a metastatic lesion was consistent with dedifferentiated chordoma. The patient was treated sequentially with Imatinib, radiation therapy, Mesna-Adriamycin-Ifosfamide, and Dasatinib (on trial SARC009) without response, and he died in March 2010.

A sample of the resected primary tumor was used to establish the xenograft. A single cell suspension of tumor cells was implanted in the parasacral region of a NOG/SCID mouse. After approximately 90 days, a visible tumor had grown (Figure 1C). The mouse was euthanized and the tumor was harvested. Fragments from the harvested tumor were implanted into additional mice for propagation. Implanted tumor fragments reliably and consistently grow upon serial implantation in NOG/SCID mice with over 12 passages since the original xenograft and over 90% of implantations resulted in tumor growth. The rate of tumor growth accelerated after the original implantation, but stabilized such that tumors now reach a diameter of 2 cm approximately 4 to 6 weeks after implantation. 

### Characterization of the xenograft

**Figure 2:**
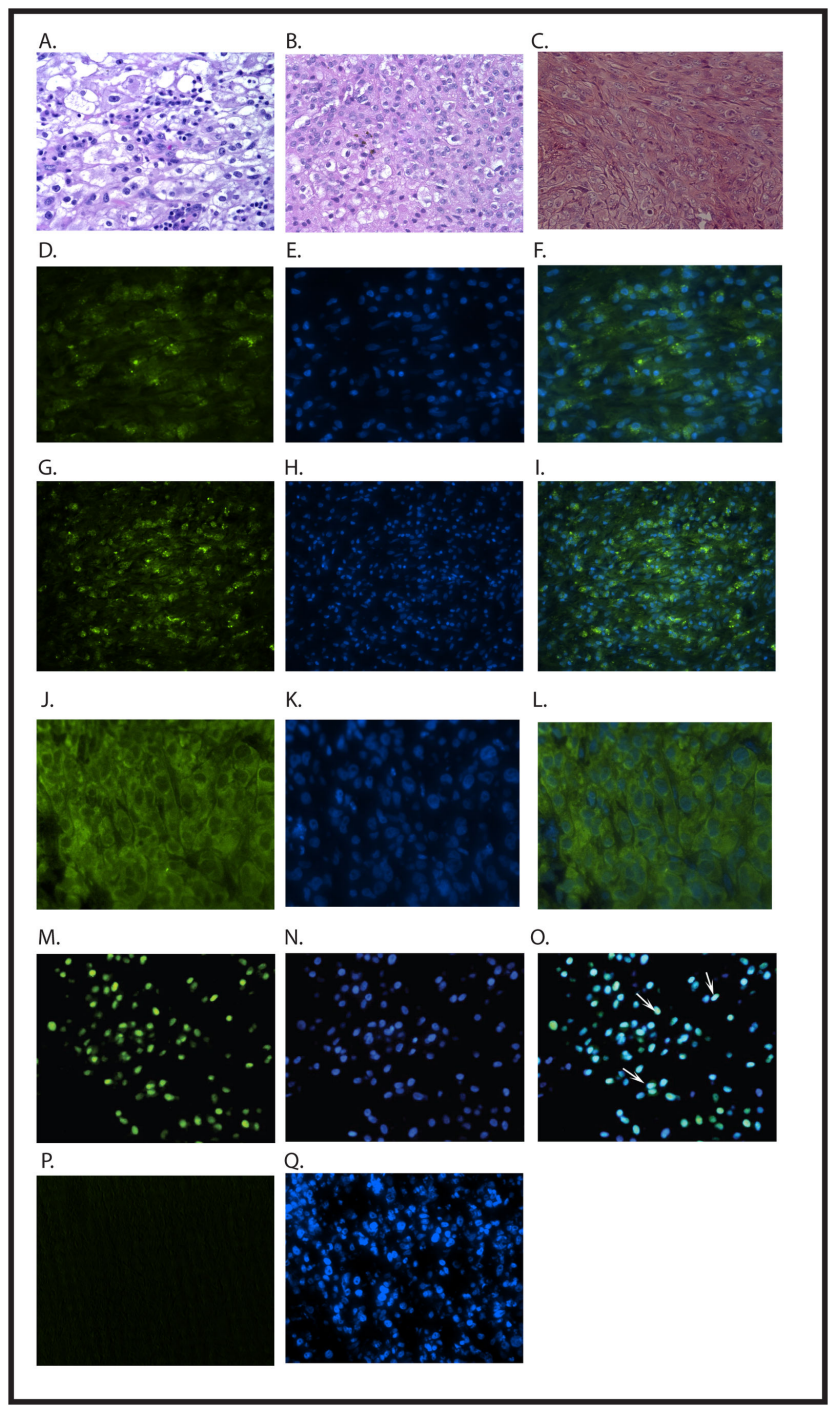
A) H&E section of patient’s primary tumor displaying classical chordoma histology. B) H&E section of patient’s metastatic relapse, with typical dedifferentiated histology. C) H&E section of xenograft showing dedifferentiated histology, similar to patient’s metastatic relapse. The staining shown in this panel is different from the staining in panels A and B because the former were performed in the clinical pathology laboratory of The Johns Hopkins Hospital, while the latter was performed in our laboratory. D-F) Immunofluoresecent evaluation of a sample from the patient’s metastatic relapse. Green staining (D) shows localization of brachyury, and DAPI (E) highlights the nuclei. The overlay (F) demonstrates the cytoplasmic localization of brachyury. These experiments were performed using the Santa Cruz anti-brachyury antibody C-19. G-L) Immunofluorescent evaluation of the xenograft. Green staining (G) shows brachyury, and DAPI (H) highlights the nuclei. Again, the overlay (I) demonstrates the cytoplasmic localization of brachyury. These experiments were performed using the Santa Cruz anti-brachyury antibody C-19. Using a second antibody, Santa Cruz anti-brachyury antibody A-4, green staining (J) shows localization of brachyury, and DAPI (K) highlights the nuclei. The overlay (L) demonstrates the cytoplasmic localization of brachyury. M-O) Immunofluorescent evaluation of an archival classic chordoma showing typical brachyury (green; M) localized to the nucleus, as identified by DAPI (blue; N), which colocalize in the overlay (O). The arrows indicate some of the nuclei with simultaneous staining for both brachyury and DAPI. To confirm the specificity of the A-4 antibody, we performed immunostaining of an osteosarcoma xenograft. No staining was seen (P) despite adequate DAPI staining (Q). These experiments were performed in parallel with staining of sections from the chordoma xenograft which yielded images identical to those shown (J-L). Images A-I are 200x, images J-L are 400x, and images M-Q are 200x.

 RNA was isolated from the xenograft and evaluated by RT-PCR, confirming the expression of brachyury (Figure 1D). Histologic evaluation of the xenograft showed dedifferentiated morphology, similar to the morphology of the patient’s metastatic relapse (Figure 2A, B, C). Immunohistochemical analysis of the xenograft also identifies strong expression of brachyury in the xenograft (Figure 2G). Interestingly, by immunohistochemistry brachyury protein was localized to the cytoplasm instead of the typical nuclear localization (Figure 2G-L). This mislocalization of brachyury was also seen in the patient’s metastatic relapse (Figure 2D, E, F). To ensure that the cytoplasmic brachyury staining was not the result of a technical problem, we evaluated a different patient’s conventional chordoma sample, and were able to demonstrate nuclear localization of brachyury in that tumor (Figure 2M-O). As an additional control, we saw no immunohistochemical staining for brachyury in an osteosarcoma xenograft (Figure 2P, Q). Thus, the cytoplasmic localization of brachyury seen in the xenograft is also seen in the original tumor from which the xenograft was derived, and does not reflect the inability of these reagents to detect nuclear brachyury protein. Despite the mislocalization, brachyury expression supports the identification of this tumor as a dedifferentiated chordoma.

**Figure 3:**
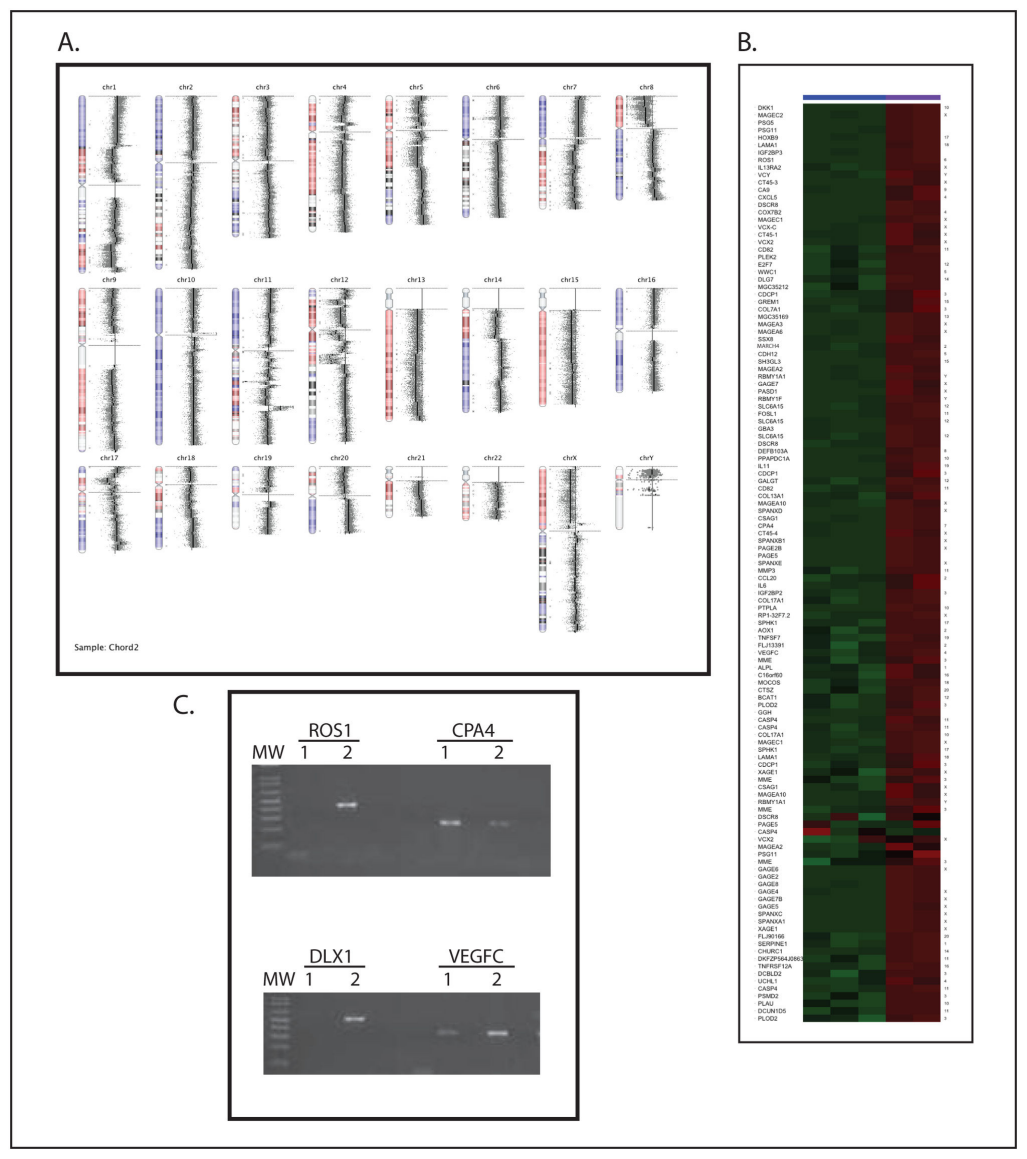
A) SNP-genotyping showing significant loss of heterozygosity throughout the genome. B) A heatmap showing relative expression of the 100 genes most strongly overexpressed in the xenograft compared with classic chordomas. Chromosome location of each gene is denoted along the right side. Green represents relative underexpression, and red represents relative overexpression. The three columns on the left are data from the comparison chordomas, and the two columns on the right are from the xenograft. C) RT-PCR confirmation of gene expression in the xenograft. cDNA reverse transcribed from RNA isolated from the xenograft and from the JHC7 cell line was amplified with primers for the indicated genes. Lanes 1 are reactions using RNA from JHC7, and Lanes 2 are reactions using RNA from the xenograft. Ribosomal RNA 36B4 was used as a positive control to confirm cDNA integrity (data not shown).

 We expanded our molecular evaluation of the xenograft by performing whole genome SNP genotyping and microarray-based gene expression profiling. We detected extensive loss of heterozygosity involving all chromosomes to a varying degree (Figure 3A), including in the region of chromosome 9p21, the locus that contains the CDKN2A/p16 tumor suppressor gene. There was no focal alteration in copy number observed at the brachyury locus (6p27) though the entire 6p arm showed loss of heterozygosity with a gain in copy number. Gene expression profiling showed numerous genes expressed at very high levels relative to 3 profiles previously generated from classic chordomas. There were 817 probes 2-fold higher than the comparison tumors with an adjusted p value < 0.01, including multiple genes coding for members of the GAGE cancer/testis antigen family (Figure 3B). In addition, a number of genes with established roles in tumorigenesis were overexpressed, including ROS1, DLX1, CPA4, and VEGFC2. We used RT-PCR to confirm the high level of expression of several of the genes seen in the microarray experiments (Figure 3C). Interestingly, a number of the genes expressed at high levels, including IL-11 [14], CXCL5 [15], VEGFC [16], and CCL20 [17] are NF-κB target genes.

### High-throughput screening of anti-chordoma drugs

**Figure 4:**
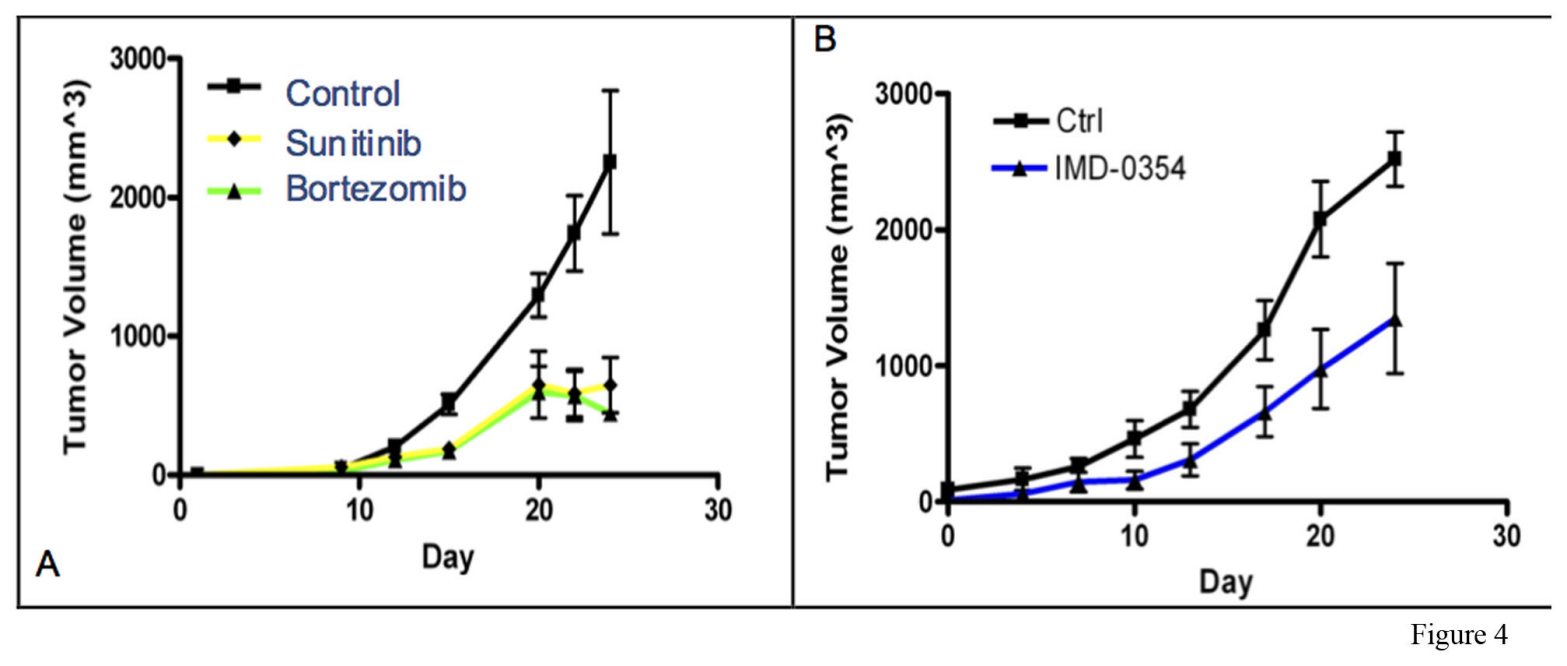
A) Chordoma xenograft tumor growth curves in control mice and mice treated with Sunitinib (p = 0.026) or Bortezomib (p = 0.015) measuring from the start of treatment. B) Chordoma xenograft tumor growth curves of control mice and mice treated with IKK-inhibitor IMD-0354 (p = 0.03) measuring from start of treatment. Each data point is the mean tumor volume, and error bars show the standard error, of the entire cohort on the indicated day. Data were analyzed for significance using a 2-way ANOVA, with p < 0.05 considered significant.

 A high-throughput screen of 2,816 approved or investigational drugs from the NCGC Pharmaceutical Collection (NPC) [12] was performed. A number of compounds were identified that demonstrated *in vitro* toxicity to the chordoma cell lines U-CH 1 and/or U-CH2B, but not the nonchordoma cell line CCL4, and these results have been separately reported [13] . To provide *in vivo* validation of this *in vitro* screen, we tested 2 of these agents, sunitinib and bortezomib, for anti-tumor activity in our xenograft model system. Both drugs significantly inhibit the growth of the chordoma xenograft in our model (Figure 4A), providing preliminary *in vivo* validation of these agents as potential chordoma treatments. 

**Figure 5:**
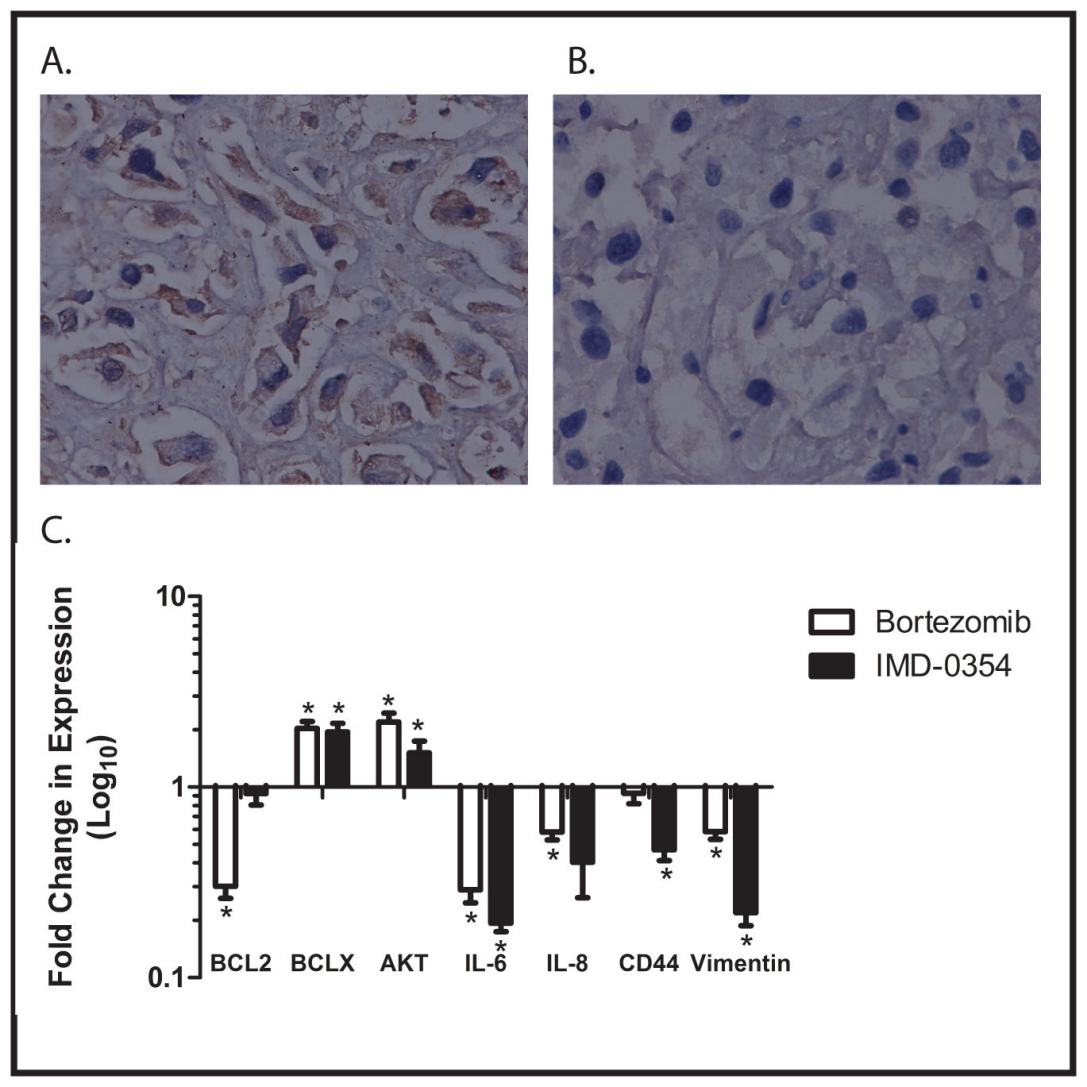
A) Immunohistochemical analysis of sections of xenograft from control mice showing expression of NF-κB p65 (brown staining). (B) Immunohistochemical analysis of sections of xenograft from mice treated with bortezomib, also stained with antibody for the p65 subunit of NF-κB. No staining is evident in these sections. Images are at 200x magnification. C) RNA was isolated from xenografts growing in untreated, control mice, and in mice treated with either bortezomib or IMD-0354. After reverse transcription, quantitative RT-PCR was performed to measure expression of the indicated NF-κB target genes. Data are presented as fold change between mice treated with the indicated agent and control. Experiments were performed in triplicate, and error bars show standard error of the mean. *=achieves statistical significance compared to control (p<0.05) by two-tailed unpaired Student t test.

Bortezomib is a proteasome inhibitor thought to act clinically by inhibiting the activity of nuclear factor κB (NF-κB) [18]. Our finding that bortezomib inhibits the growth of the xenograft therefore may implicate NF-κB in the regulation of proliferation and growth of dedifferentiated chordoma, prompting further investigation of the role of NF-κB in the biology of our xenograft. To confirm that bortezomib is affecting NF-κB in this system, we performed an immunohistochemical analysis of expression of the p65 subunit of NF-κB in control xenografts and in those treated with bortezomib. Expression of p65 was detected in control tumors but not in tumors treated with bortezomib (Figure 5A), supporting the hypothesis that bortezomib inhibits tumor growth through inhibition of NF-κB activity. 

To provide further support for the importance of NF-κB in the biology of dedifferentiated chordoma, we investigated the effect of other NF-κB inhibitors on xenograft growth. Canonical NF-κB activation results from activation of the IKK kinase, leading to IκB phosphorylation and subsequent proteasomal degradation, releasing the p65 subunit of NF-κB to enter the nucleus and recruit co-activators such as p300 to NF-κB target genes. Bortezomib, by inhibiting proteasomal degradation of IκB, prevents nuclear translocation and target gene activation. A complementary way to block this pathway is through the use of IKK inhibitors, such as IMD-0354 [19]. We therefore treated mice implanted with the xenograft with either IMD-0354 or vehicle control. Administration of IMD-0354 significantly inhibited xenograft growth *in vivo* (Figure 4B), strongly supporting the importance of NF-κB in regulating the growth of dedifferentiated chordoma. 

As a final confirmation that bortezomib is functioning by inhibiting NF-κB, we investigated the expression of a panel of NF-κB target genes in control tumors and tumors treated with either bortezomib or IMD-0354. Quantitative RT-PCR demonstrated that expression of the known NF-κB target genes Bcl-2, IL-6, IL-8, CD44, and vimentin was significantly decreased in tumors treated with either drug compared with control (Figure 5C). Interestingly, 2 other target genes, Bcl-x and AKT were actually upregulated in drug treated tumors compared with control. Nevertheless, these findings strongly implicate NF-κB as an important regulator of the growth of dedifferentiated chordoma.

## Discussion

Several new chordoma cell lines and xenograft models have been recently developed, which will markedly improve the ability to study this rare tumor. Three groups to date have established chordoma cell lines from primary chordoma samples and have been able to inject the cells into immunocompromised mice and establish tumors [10,20,21]. These xenografts, however, are not serially transplantable, though the cell lines from which they were derived can be passaged *in vitro*. More recently, Siu et al. reported the establishment of the first primary chordoma xenograft [22], which was established directly from samples obtained from a patient’s primary tumor resection and had not been grown *in vitro* prior to implantation. It displays classical chordoma histology, is serially transplantable, has a “take rate” of 93%, and tumor growth is in the range of approximately 100 days per passage. We contribute to this growing arsenal of chordoma model systems by describing the establishment of the first dedifferentiated chordoma xenograft and demonstrating its use to test therapeutic agents *in vivo*. In doing so, we have identified NF-κB signaling as a key pathway in the growth of chordoma. 

Though the tumor from which our xenograft was derived was originally classified as a classical chordoma, our xenograft demonstrates dedifferentiated histology. The patient’s metastatic relapse also demonstrated dedifferentiated histology. It has been suggested that classical chordomas can include pockets of dedifferentiated tissue [22] and we suspect that the sample we obtained from the patient’s tumor resection may have contained dedifferentiated elements. Although no further tissue is available from the primary resection, histological and immunohistochemical analysis reveals similarities between the xenograft and tissue from the metastatic relapse. We demonstrated brachyury expression, thought to be a specific marker for chordoma [23], in our xenograft both by PCR and by immunohistochemistry. Brachyury is mislocalized to the cytoplasm in our xenograft, and this aberrant expression is also seen in pathologic sections from the relapsed tumor. We demonstrated cytoplasmic brachyury staining in the xenograft using 2 distinct antibodies, but were able to demonstrate nuclear localization of brachyury in a sample of classical chordoma using these antibodies, making it highly unlikely that this finding is a technical artifact. Mislocalization of transcription factors to the cytoplasm has been reported in other tumors, usually with significant functional consequences. For example, RUNX3 is a transcription factor with tumor suppressor activity that is mislocalized to the cytoplasm in breast cancer [24]. Nuclear expression in breast cancer cell lines results in decreased invasiveness *in vitro* and decreased tumorigenesis *in vivo*, suggesting that cytoplasmic localization prevents RUNX3 from acting as a tumor suppressor. Similarly, cytoplasmic localization of the transcription factor FOXF1 is positively associated with histologic grade, depth of invasion, and lymphatic metastasis of colorectal carcinoma [25]. Localization to the cytoplasm might inhibit the ability of brachyury to drive a differentiation program in the tumor cells, thus it is interesting to speculate that mislocalization of brachyury to the cytoplasm may be causally related to the dedifferentiated phenotype exhibited at relapse. Whether cytoplasmic localization of brachyury is unique to this patient’s tumor or is a previously unrecognized feature of dedifferentiated chordoma, and the implication of this finding to the pathogenesis of this tumor, all remain open questions. 

Molecular analysis of our xenograft both supports the diagnosis of dedifferentiated chordoma and reveals some novel aspects of chordoma pathogenesis. SNP genotyping revealed extensive loss of heterozygosity throughout the genome. Among the affected sites is chromosome 9p21, one of the more common deletions seen in chordomas [26]. The loss of the CDKN2A/p16 tumor suppressor gene located in this region may play an important role in chordoma tumorigenesis. Furthermore, SNP genotyping showed moderate copy number loss of the IKB-alpha gene (14q13). IKB-alpha is one of the major inhibitors of the NF-κB pathway. The loss of the suppressive effects of IKB-alpha may potentiate NF-κB signaling. The combination of loss of CDKN2A/p16 and activation of NF-κB has been reported in other tumor types and may be significant for the formation of chordoma as well [27]. In addition to SNP genotyping, gene expression profiling showed upregulation of numerous genes coding for members of the GAGE cancer/testis antigen family. These antigens are integral to immune-privileged sites, such as the testis, and are believed to be a method by which certain tumors escape immune surveillance [28]. More work will be needed to determine how common GAGE antigen expression is in chordoma, something that will be important to understand as efforts to develop anti-chordoma immunotherapy advance. 

*In vitro* drug screening is an important tool in developing new treatment plans for rare tumors. One limitation to this approach, however, is that many drugs that appear to be active *in vitro* show no activity *in vivo*. In the case of chordoma, a second limitation is that drugs which show activity against one subtype may not be active against other subtypes. Thus, it is critical to validate *in vitro* results in an animal model prior to initiating clinical trials in humans. This is especially true in a rare disease like chordoma, because if only limited numbers of patients are available for clinical trials, there must be a high degree of certainty that a drug will be effective before a trial can be initiated. Our chordoma xenograft offers some important benefits in this regard, compared with other established models: 1) the relatively rapid tumor growth (4 to 6 weeks, compared with 3 to 6 months for other xenografts), 2) the xenograft is from a primary tumor, not from cell lines adapted to growth in tissue culture prior to implantation, and 3) the dedifferentiated histology complements the classical histology of established cell lines, allowing identification of agents that might be effective regardless of tumor histology. Our high-throughput screen was performed using the U-CH1 and U-CH2B cell lines, which were derived from classical chordomas, and our confirmation of *in vivo* activity with a dedifferentiated chordoma xenograft implies that these agents may have broad utility in chordoma patients. Our finding that sunitinib has activity *in vitro* and *in vivo* provides some validation of this approach, as this drug has been used to treat several chordoma patients with evidence of efficacy [29]. 

As discussed above, there is still only a very limited understanding of the signaling pathways important in chordoma biology. Our finding that bortezomib is effective both *in vitro* and *in vivo* is novel and implicates NF-κB signaling in the pathogenesis of this tumor, also a novel finding. Supportive of the importance of NF-κB signaling in the pathogenesis of this tumor was our finding that 4 NF-κB target genes, IL-11, CXCL5, VEGFC, and CCL20, are on the list of strongly expressed genes from our gene expression profiling analysis. To confirm that bortezomib’s activity against chordoma is due to inhibition of NF-κB, we also tested the activity of the IKK inhibitor, IMD-0354, in our chordoma xenograft. IKK is essential for allowing nuclear translocation of NF-κB. By inhibiting IKK, IMD-0354 does not allow NF-κB to enter the nucleus and activate transcription of target genes. We found that IMD-0354 also inhibits the growth of chordoma, and demonstrate inhibition of a number of NF-κB target genes in treated xenografts, strongly supporting the importance of NF-κB in the growth of this tumor. Interestingly, 2 NF-κB target genes with specific anti-apoptotic activity were overexpressed in tumors treated with either bortezomib or IMD-0354 compared with control. At the time that tumors were harvested for analysis, they were growing despite drug treatment, suggesting that therapeutic resistance had developed. Overexpression of Bcl2 and AKT at that time point suggests a mechanism of resistance. Interestingly, activation of AKT has previously been implicated in the biology of chordoma through an analysis of primary patient samples [5]. The fact that this gene expression pattern is seen in tumors treated with both anti-NF-κB agents suggests that this mechanism of resistance may be generalizable, and implies that combination therapy with agents that block Bcl2 and/or AKT may result in additive or even synergistic anti-tumor activity, and this will be investigated in future work.

In summary, we report here the establishment of the first dedifferentiated chordoma xenograft. This new model system was used to provide *in vivo* validation of a high throughput *in vitro* drug screen for new agents with activity against chordoma, the results of which strongly implicate NF-κB as a critical mediator of chordoma growth. The combination of *in vitro* screening with conventional chordoma cell lines and *in vivo* verification of activity using a rapidly growing dedifferentiated chordoma xenograft is a powerful approach to both drug discovery and to understanding the biology of this rare tumor.
